# From Benchtop to Bedside: A Review of Oncolytic Virotherapy

**DOI:** 10.3390/biomedicines4030018

**Published:** 2016-08-02

**Authors:** Audrey H. Choi, Michael P. O’Leary, Yuman Fong, Nanhai G. Chen

**Affiliations:** 1 Department of Surgery, City of Hope National Medical Center, Duarte, CA 91010, USA; audreychoi@gmail.com (A.H.C.); moleary@coh.org (M.P.O.); yfong@coh.org (Y.F.); 2 Center for Gene Therapy, Department of Hematology and Hematopoietic Cell Transplantation, Beckman Research Institute, City of Hope National Medical Center, Duarte, CA 91010, USA

**Keywords:** oncolytic viruses, immunotherapy, virotherapy

## Abstract

Oncolytic viruses (OVs) demonstrate the ability to replicate selectively in cancer cells, resulting in antitumor effects by a variety of mechanisms, including direct cell lysis and indirect cell death through immune-mediate host responses. Although the mechanisms of action of OVs are still not fully understood, major advances have been made in our understanding of how OVs function and interact with the host immune system, resulting in the recent FDA approval of the first OV for cancer therapy in the USA. This review provides an overview of the history of OVs, their selectivity for cancer cells, and their multifaceted mechanism of antitumor action, as well as strategies employed to augment selectivity and efficacy of OVs. OVs in combination with standard cancer therapies are also discussed, as well as a review of ongoing human clinical trials.

## 1. Introduction

Oncolytic viruses (OVs) represent an emerging class of cancer therapeutics, poised at the junction of biologic therapy and immunotherapy. Whether wild-type or genetically-modified, OVs have the ability to selectively replicate in cancer cells, causing antitumor effects through a variety of mechanisms, including direct lysis of infected cells and immune-mediated destruction of both infected and non-infected cells [1,2,3].

Clinicians noted the antitumor effect of naturally occurring viral infection in cancer patients over 100 years ago [4,5,6]. However, only recent advances within the last 25 years in the understanding of how OVs function and interact with the immune system have been able to deliver OVs into clinical trials for humans. In October 2015, a herpes simplex virus 1 (HSV-1) expressing granulocyte-macrophage colony-stimulating factor (GM-CSF) named T-VEC (talimogene laherparepvec, Amgen, Inc., Thousand Oaks, CA, USA) became the first OV approved by the US Food and Drug Administration (FDA) for the treatment of advanced melanoma [7]. Although oncolytic adenovirus H101 had already been approved by China’s State Food and Drug administration in 2005 [8], T-VEC’s FDA approval was undoubtedly a great milestone for the field of oncolytic virotherapy, thereby establishing OVs as a new class of cancer therapeutic agents for use in the US [9].

In this review, the mechanisms of natural viral tropism for cancer cells and antitumor effect will be discussed in detail, as well as the numerous strategies that have been used to augment OV selectivity and efficacy against cancer cells. Additionally, OVs in combination with chemotherapy, radiation therapy and immunotherapies are reviewed, particularly in the context of ongoing clinical trials.

## 2. Brief History of Oncolytic Viruses

Although viruses have been utilized as therapeutic agents in the form of vaccines since the late 1700s [10,11], their potential application as a cancer therapy had not been explored until a series of case reports dating back to the early 1900s chronicled several cancer remissions after concurrent infection with naturally-acquired viral illnesses [4,5,6]. In the most often-cited report, G. Dock describes the case of a 42-year old woman suffering from leukemia who experienced remission after infection with presumed influenza [5]. In another case, a four-year old boy with leukemia demonstrated a remarkable remission after acquiring chickenpox [4]. Unfortunately, after a one-month remission, his leukemia relapsed and progressed rapidly to death.

Several early landmark human clinical trials highlighted both the potential of viral therapy as a cancer treatment, as well as its dreadful side effects [12,13,14,15]. Although some patients experienced short-lived clinical remission of their cancers, a notable proportion either died from the side effects of the viral therapy (hepatitis or fatal neuroencephalitis) [14,15] or had the brief remissions that were reversed by a strong anamnestic response by the patient’s immune system, leading to continued cancer progression and death from the primary disease [12,13].

These observations suggested that naturally occurring viruses possessed an innate ability to kill cancer, but in order to harness the potential of oncolytic viral therapy, alterations would be required to both improve cancer cell selectivity and efficacy. With the advent of cell culture in the 1950s [16], virus research in the in vitro setting advanced rapidly, as did the development of animal models, pioneered by Alice Moore. Moore first demonstrated that Russian Far East encephalitis virus could completely shrink mouse sarcoma [17]. Despite the fact that many mice died from fatal encephalitis, the findings were a landmark proof of principle.

Following a series of disappointing clinical trials, interest in oncolytic viruses waned in the 1970–80s until the development of genetic engineering in the 1990s made it possible to alter viral genomes [18]. This technological advancement allowed manipulation of the viral genome to improve selectivity and decrease toxicity. In another landmark study, Martuza and colleagues reported that treatment with a thymidine kinase (TK)-mutated HSV-1 could shrink glioma in mice brains with reduced neurotoxicity [19].

Since then, the development of viral therapy from laboratory to bedside has finally been realized. ONYX-015 became the first virus to enter Phase I clinical trials in 1996 and the adenovirus mutant H101 became the world’s first OV approved for cancer treatment in 2005 [8]. Following a Phase III trial showing improved durable response rate for the intralesional treatment of melanoma, T-VEC became the first oncolytic virus approved by the FDA in October 2015 [7].

## 3. Mechanisms of Tumor Selectivity

### 3.1. Natural Viral Tropism for Cancer Cells

Some viruses demonstrate a natural ability to target cancer cells selectively and more efficiently than normal cells. Cancer cells have several distinct hallmarks that separate them from normal cells: sustained growth signals, insensitivity to anti-growth signals, evasion of apoptosis, increased angiogenesis, cell immortality, and invasion/metastasis [20,21]. Strikingly, cells infected with viruses demonstrate many of the same properties as transformed cancer cells, as viruses have evolved various mechanisms to replicate within the host while evading detection. In fact, some viruses naturally exploit the aberrant signaling pathways that maintain sustained cancer growth in order to selectively infect and replicate within cancer cells as opposed to normal cells. For instance, constitutively active AKT pathway signaling serves as a sustained growth and survival signal in many different types of cancer [22]. Wang and colleagues demonstrated that the natural tropism of myxoma virus in cancer cells capitalizes on endogenous AKT activity via complex formation between AKT and M-T5, a myxoma viral protein [23].

Cancer cells have been shown to overexpress selected surface receptors, which is another mechanism by which viruses may selectively bind to and infect cancer cells. In squamous cell carcinoma, higher expression of the cell surface adhesion molecule nectin-1 correlated with increased HSV-1 infection and cytotoxicity compared to cells that had lower nectin-1 levels (Table 1) [24]. Measles virus has been shown to utilize the surface receptor CD46 for cellular entry, which is overexpressed in a variety of human cancers, including hepatocellular carcinoma, colorectal cancer, ovarian cancer, and breast cancer (Table 2) [25,26].

In addition, OVs can also capitalize on deficient anti-viral defense mechanisms in cancer cells. When normal cells are infected by viruses, release of interferons (IFNs) and activation of toll-like receptors (TLRs) by recognition of viral elements activate several downstream pathways, leading to protein kinase R (PKR) activation [27]. Phosphorylated PKR subsequently blocks protein synthesis and prevents viral replication in the cell. Cancer cells may have abnormal IFN pathways and/or abnormal PKR activity, making them more susceptible to viral infection. OVs have also exploited the differences in cancer cell pathways involving retinoblastoma (RB), epithelial growth factor receptor (EGFR), Ras, and Wnt, which have been reviewed in detail previously [28,29].

### 3.2. Enhancing OV Tumor Selectivity

Viral genome modification has been an important strategy to enhance tumor selectivity since the advent of genetic engineering in the 1990s. There are two general points of intervention that can be thought of when designing strategies to enhance tumor selectivity: (1) targeting selectivity of viruses prior to entering a cancer cell (transductional targeting); and (2) controlling selectivity of replication once the virus has infected a cell (viral gene inactivation, transcriptional targeting, microRNA targeting sequences).

Restricting viral entry and infection through transductional targeting has been accomplished by several methods, including the use of adaptor molecules to facilitate binding of viral attachment proteins to a specific target cell’s receptor, pseudotyping with a different viral strain’s attachment protein, and genetically engineering viral expression of target ligands directed at specific tumor cells [18]. These strategies have been applied to increase the tumor selectivity of oncolytic adenoviruses, vaccinia virus (VACV), and measles virus [30,31,32].

However, the focus of this section will be on the various methods employed to control selective viral replication within cancer cells, thereby limiting viral replication in normal cells. Viral gene inactivation is a commonly used strategy to limit viral infection in cancer cells, often capitalizing on alterations in cellular metabolism and survival pathways in transformed cells. For example, both ONYX-015 and H101 have deletions in the adenoviral protein gene for E1B 55K, which normally inactivates the tumor suppressor p53. Originally it was thought that when these E1B 55K-negative viruses infect normal cells, their defective E1B 55K proteins could not block the cell’s normal apoptotic defense mechanism, limiting viral infection in non-cancer cells [33,34]. However further studies have challenged this hypothesis and suggested that the mechanism of selectivity is based on late viral mRNA transport [35]. Another example is deletion of the gene that encodes TK in both VACV and HSV-1. Since TK is required for DNA and RNA synthesis, deletion of viral TK leads to the virus’ dependence on the host cell’s TK activity, which is more pronounced in cancer cells compared to normal cells [36]. TK-deleted versions of VACV and HSV-1 have been demonstrated to be less pathogenically virulent, while still having potent antitumor effect in vivo [19,37,38,39].

Transcriptional targeting is another method that is used to produce tissue-specific OV replication by putting viral essential genes under the control of desirable promoters. The CV706 adenovirus construct was designed with the E1A viral protein (essential for viral replication) under control of the prostate-specific antigen (PSA) promoter. Since prostate cancer cells express higher levels of PSA more highly than normal cells, E1A is selectively expressed in these cells, resulting in viral replication and eventual oncolysis [40]. However, normal cells, which do not express PSA at high levels, will not generate significant amounts of E1A, resulting in defective viral replication and, thus, sparing healthy tissue from lysis.

More recently, microRNA (miRNA) targeting sequences have been used to facilitate viral selectivity for tumor cells. Interestingly, miRNA are small non-coding RNAs that are often expressed in a tissue-specific manner [41]. They can regulate post-transcriptional gene expression by binding to the 3′ untranslated region (UTR) of mRNA and preventing them from being translated. Viral genomes have been engineered to express a variety of miRNA targeting sequences (miRNA-TS) that can be designed to a specific tissue. After a cell has been infected, viral RNA is produced and transported to the cytoplasm with the miRNA-TS present. If the cell expresses the cognate miRNA, then the miRNA-TS is bound and targeted for degradation, preventing translation of the viral protein product [42]. This strategy takes advantage of the fact that miRNAs are differentially expressed in normal cells (high) and cancer cells (low), due to cancer cells’ expression of short hairpin RNA (shRNA) that target components of miRNA processing machinery. This results in a global decrease in mature miRNAs in the cancer cell and a more pronounced invasive phenotype [43,44]. One example is the let-7a miRNA, a tumor suppressor miRNA that is expressed in low levels in cancer cells, but in high levels in normal cells. A genetically-modified vesicular stomatitis virus (VSV) containing three copies of the let-7a miRNA target sequence in the 3′ UTR of the essential viral gene *M* has been shown to target tumor cells in vivo, while reducing pathologic infection of normal cells [45]. Tissue-specific differences in miRNA levels have been used to target measles virus replication to glioma cells by using neuron-specific miR7, as well as to limit unwanted side effects of viral therapy, such as hepatotoxicity in adenoviral therapy by incorporating the hepatocyte-specific miR122 target sequence in the adenoviral genome [46,47].

## 4. Mechanisms of Action

### 4.1. Intrinsic Mechanisms

Although the mechanisms of action of oncolytic viruses are still incompletely understood, it appears that the overall antitumor effect induced by oncolytic viral treatment has two major components: (1) local cell death of both virally-infected and non-infected cancer cells; and (2) induction of the systemic immune response to virally-induced cell destruction within the tumor.

OV infection of a cancer cell results in cell death by multiple mechanisms, including apoptosis, pyroptosis (caspase-1-dependent cell death), autophagic cell death, and necrosis, which is often dependent on either the virus type, the cancer cell type or a combination of both [48,49,50]. OV-mediated cell death releases cytokines, tumor-associated antigens (TAAs), and other danger signals, including damage-associated molecular pattern molecules (DAMPs) and pathogen-associated molecular pattern (PAMPs) molecules. The host immune response to these signals has been associated with local release of cytotoxic perforins and granzymes that can kill adjacent non-virally infected tumor cells, the so-called “immune-associated” bystander effect [51,52,53,54,55]. Additionally, some types of OVs also target tumor vasculature, leading to death of uninfected tumor cells due to loss of the tumor blood supply [56,57].

Perhaps with the exception of apoptosis, the remaining modalities of cell death listed above are highly immunogenic, leading to activation of both the innate and adaptive immune responses. Direct oncolysis of virus-infected cancer cells leads to release of TAAs, which function as weak antigens and can include mutated proteins, fusion proteins, and tissue- and/or cancer-specific overexpressed proteins [52]. When the host immune system is activated and primed against TAAs, antitumor effects due to cytotoxic CD8+ T cell activation can be observed at distant tumor sites that were not locally treated with the virus [58]. In addition to releasing OV-specific PAMPs, virally-mediated cell death by necrosis and autophagy also release DAMPs, including adenosine triphosphate (ATP), calreticulin, heat shock proteins (HSPs), and high mobility group box 1 (HMGB1) protein [53,54,55,59,60]. Lastly, dying cells also release a variety of cytokines into the local environment, such as interferons (IFNs), tumor necrosis factor-alpha (TNF-α) and interleukins (IL), that promote further cell-mediated immune response [61,62,63]. Taken together, the presence of TAAs, PAMPs, DAMPs and cytokines stimulate antigen presenting cell (APC) maturation which, in turn, primes both CD4+ and CD8+ T lymphocytes in the adaptive host immune response by cross-presentation [48,64,65,66,67]. Moreover, type I IFNs and DAMPs can also directly stimulate natural killer (NK) cell response against cancer cells, as one example of how the innate immune system is also involved in the antitumor response after OV treatment [27].

Notably, cell death mediated by OVs seems to receive a significant contribution from neutrophils and has been reported and reviewed elsewhere [68,69,70]. In fact, often observed following therapy, neutrophils could be even more important than CTLs in driving OV-mediated cell death [69,71,72]. Neutrophils are vital immune first responders, being thus responsible for initiating an antimicrobial response at sites of infection [73]. Neutrophils play important roles in viral pathogenesis and, therefore, not surprisingly are involved in responses to OVs. Once activated, in addition to secreting the TNF-related apoptosis inducing ligand (TRAIL) and TNF-α, neutrophils also generate large quantities of reactive oxygen species (ROS), which can cause not only the destruction of target microorganisms, but also disseminated cell death (necrosis/necroptosis), contributing to further inflammation and to the oncolytic effect [69,73].

It is important to note, however, that although the multimodal immunogenic cell death mediated by OV infection is able to activate the host immune system effectively against tumor cells, the same process can be detrimental to the continued propagation of OVs. The systemic antitumor response can result in clearance of OV by antibodies generated against viral PAMPs and/or cytotoxic T cells that recognize viral PAMPs [27,74]. NK cells have also been directly implicated in decreasing the efficacy of viral therapy via upregulation of natural cytotoxicity receptors in virally-infected cells [75].

### 4.2. Enhancing OV Antitumoral Response

Just as deletion of viral genes has been used to improve tumor selectively, insertion of therapeutic genes via genetic engineering has been employed to enhance OV antitumoral responses. One major strategy that will be the focus of this section is the expression of immune-stimulating molecules, including (1) cytokines; (2) molecules that enhance immune system cross-priming; (3) T lymphocyte co-stimulatory molecules; and (4) chemokines.

A number of previously studied OVs expressing cytokines have demonstrated promising results. Several OVs currently in clinical trials express GM-CSF, a cytokine that promotes APC maturation and stimulates cytotoxic T lymphocyte (CTL) responses against tumors. T-VEC is the most studied OV expressing GM-CSF. Analysis of biopsies from melanoma lesions treated by intratumoral T-VEC injection induced both local and systemic T lymphocyte responses, as well as decreased CD4+FoxP3+ regulatory T cells (Tregs) and CD8+FoxP3+ suppressor T cells (Ts), both of which are inversely correlated with patient survival [76]. The GM-CSF-expressing poxvirus JX-594 (pexastimogene devacirepvec; Pexa-Vec) has been tested in several Phase I/II trials and shown to induce antibody-mediated, complement-dependent cancer cell cytotoxicity in a variety of solid tumors in humans [77]. Other OVs armed with GM-CSF that have been evaluated in clinical trial include Oncos-102 (adenovirus), CG0070 (adenovirus), and OrienX010 (HSV) [78,79,80]. OVs have also been armed with other cytokines, such as IL-2, IL-12, IL-15, and IL-18, in an attempt to enhance NK cell response and CTL activity [81,82,83,84].

Heat shock proteins (HSPs) are released during oncolysis of OV-infected tumor cells, leading to induction of chemokine production and activation of dendritic cells via the TLR4 pathway [85]. Given that HSP release cross-primes the innate and adaptive immune systems, OVs were engineered to overexpress HSPs, in particular HSP70. Adenoviral vectors expressing HSP70 have demonstrated antitumor effect in patient-derived xenograft models of hepatocellular cancer (HCC), as well as in a Phase I trial of solid tumors in humans [86,87].

Directly targeting T lymphocyte activation by engineering OVs that express T lymphocyte co-stimulatory molecules is another approach aimed at increasing CTL activation against tumor cells. Professional APCs possess co-stimulatory molecules such as CD40 and B7-1. When CD40L (CD154), a transmembrane protein expressed on CD4+ T cells, binds to its receptor on an APC, a T-helper (Th1) response is induced and leads to CTL activation. Along similar lines, binding of CD28 on T lymphocytes to B7-1 triggers CTL proliferation and activation. Preclinical models using CD40L- and B7-1-armed OVs have shown induction of Th1 response and antitumor effects [2,88,89].

Although multiple strategies designed to increase activation of APCs and CTLs have been tested, the ability of these activated CTLs to effectively home to and destroy tumor cells may be one barrier to OV efficacy. By engineering OVs that express selected chemokine ligands, investigators are altering the local tumor microenvironment in OV-infected tissue in order to increase migration of appropriately primed CTLs in solid tumors. For example, OVs have been created that express CCL5/RANTES, whose receptor is expressed on CTLs. An adenovirus expressing CCL5/RANTES increased chemotaxis of NK92 cells in vitro, as well as exerted antitumor effect in an HCC nude mouse xenograft model [90]. VACV expressing CCL5/RANTES and CXCL11 have both been shown to increase local trafficking of CTLs and reduce tumor volume in virus-treated mouse models [91,92]. Furthermore, OV-induced expression of chemokines is being studied as an exciting adjunct to chimeric antigen receptor (CAR)-T cell development for solid tumors. CAR-T cells, which combine the antigen recognition properties of a monoclonal antibody with the constant region of a T cell receptor, can identify and kill tumor cells irrespective of the major histocompatibility complex (MHC) [93]. This constitutes an advantage over native MHC-restricted CTLs, as tumor cells have developed strategies to evade the immune system, such as downregulation of human leukocyte antigen (HLA) class I molecules and defective antigen processing [94]. Although CAR-T cells have had success in treating hematologic malignancies, the efficacy in solid tumors has not been as effective due to poor CAR-T cell penetration. Therefore, the potential to combine chemokine-expressing OVs with CAR-T cells has generated considerable interest and is currently being studied in preclinical models [93,95,96].

Lastly, there are several non-immune-directed strategies of increasing OV efficacy that warrant a brief review: (1) suicide genes; (2) anti-vasculature molecules; (3) extracellular matrix-targeting molecules; (4) fusogenic membrane protein expression; and (5) strategies to prevent viral clearance or inactivation. Expression of suicide genes makes cells more susceptible to apoptosis or treatment with other drugs. Therefore, inclusion of suicide genes on viral vectors has been utilized to increase cancer susceptibility to OV treatment. For example, TNF-α and TRAIL are two pro-apoptotic molecules engineered on viral constructs to enhance OV efficacy [97,98]. Included suicide genes can also encode enzymes that convert prodrugs into active drugs, thereby increasing targeted drug delivery when expressed by an OV-infected cell [99]. An adenovirus containing the bacterial enzyme cytosine deaminase (which converts the prodrug 5-fluorocytosine to cytotoxic 5-fluorouracil) was created for the treatment of prostate cancer [100].

In order to induce tumor necrosis and cell death, several groups have included genes encoding anti-angiogenic molecules into their engineered viral vectors. VACV and adenoviruses have been constructed to express both anti-vascular endothelial growth factor (VEGF) single-chain antibodies and the extracellular domain of the vascular endothelial growth receptor (VEGR) to neutralize soluble VEGR, thereby preventing it from binding to the VEGFR2 receptor and stimulating angiogenesis [101,102,103].

The last three strategies are aimed at enhancing OV efficacy by increasing viral penetrance into the target tissue. In solid tissue, OV spread can be limited by extracellular matrix components (ECM) intervening between cancer cells, interstitial pressure within the tumor, and the relatively acidic and hypoxic environment of the tumor. While intratumoral co-administration of ECM-degrading compounds, such as collagenase and hyaluronidase, have been shown effective [104,105], OVs expressing hyaluronidase and matrix metalloproteinases (MMP) have also shown promise in facilitating viral spread and tumor regression [106,107,108]. In order to overcome the physical barriers of pressure, pH, and low oxygen tension within tumors, OVs have also been armed and tested with fusogenic membrane glycoproteins that allow the formation of syncytia to propagate viral infection between cells without relying solely on extracellular propagation [109,110,111].

Finally, measures to prevent viral clearance and/or inactivation have been developed and recently demonstrated to enhance OV efficacy by prolonging the presence of OV agents and, consequently, their access to tumor cells [18]. Perhaps the most novel approach has been the coupling of CAR-T cells with OVs to help protect OVs from being cleared too quickly from the body [93,96]. In contrast to typical methods of OV delivery (intravenous or intratumoral inoculation), CAR-T cells can serve as carriers, facilitating the delivery of OVs directly to tumors, once T cells freely travel throughout the body. Since OVs circulate via the carrier cells, the likelihood of being inactivated (for example by pre-formed antibodies) while in the bloodstream is greatly diminished [96].

## 5. Combination Therapies

Chemotherapy and/or radiotherapy are currently the standard of care for many malignancies, but clinical data shows that combining OVs with these systemic therapies can augment the response seen with either therapy alone. The clinical trial for the ONYX-015 showed 65% response rate in patients receiving the virus plus 5-FU/cisplatin compared to just 14% response rate for virus alone [112]. The mechanism underlying this synergistic effect between OVs and chemotherapy is not completely understood. Some chemotherapeutic agents may upregulate cell surface receptors that viruses use to enter and infect tumor cells. For example, MAP/ERK kinase (MEK) inhibitors have been shown to upregulate CAR expression, which enables enhanced adenovirus entry [113]. Other lines of evidence suggest that chemotherapeutic agents can also enhance OV function by affecting the immune response to infection [114]. Paclitaxel upregulates MHC class I molecule expression, leading to enhanced antigen presentation and immune system cross-priming [115]. Doxorubicin has been shown to downregulate PD-L1, which plays a role in immune suppression [116].

OV combination therapy with radiation has also been shown to improve antitumor response in preclinical models by enhancing apoptosis in the combination therapy [117,118]. Intratumoral HSV-1 (G207) has been used successfully in clinical trial in combination with radiation therapy for patients with recurrent glioblastoma multiforme (GBM), with six out of nine patients demonstrating stable disease or partial response [119]. Furthermore, OVs are being engineered with therapeutic genes that enhance local radioactive particle delivery, in particular radioactive iodine. Both VACV and measles virus have OV strains developed carrying the human sodium iodide symporter (hNIS), which allows entry of radioactive iodine into virus-infected cells to produce further tumor destruction through local radiation exposure [120,121,122].

Perhaps the most promising combination with OVs is with T cell checkpoint inhibitors. As previously discussed, OVs induce antitumor response by cross-priming APC cells and by activating T cells. However, recent discoveries in cancer immunotherapies have shown that induction of T cell response alone is not sufficient for sustained antitumor effect. Instead, they point out that suppression of T cell inhibitory mechanisms by blockade of T cell checkpoint factors, such as cytotoxic T lymphocyte antigen 4 (CTLA-4) and programmed death 1 (PD-1), can be useful in light of the immunosuppressive nature of advanced tumors [123]. Several current clinical trials are investigating the efficacy of OV combinations with ipilimumab, a monoclonal antibody against CTLA-4, and with pembrolizumab, a monoclonal antibody against PD-1. Recently published data from a Phase IB trial of T-VEC in combination with ipilimumab for melanoma demonstrated that combination therapy appeared more effective than either treatment alone with a good safety profile [124]. Additionally, adenovirus and measles virus vectors have been engineered to express monoclonal antibodies against CTLA-4 and PD-1 with promising results [123,125,126].

## 6. Clinical Trials

Several ongoing clinical trials are testing the safety and efficacy of OV agents alone or in combination with chemo- and/or radiotherapy. A broad range of OVs is on those trials, including OVs based on measles and VSV viral backbones. For instance, a strain of measles virus encoding the sodium iodide symporter (MV-NIS) was developed by Russell and colleagues at the Mayo Clinic (Rochester, MN, USA, 2004), engineered to express the human thyroidal sodium iodide symporter (NIS), in a way that in vivo viral infection could be noninvasively monitored by radioiodine single-photon emission computed tomography (SPECT)—computed tomography (CT) imaging [120]. In 2014, Russell and colleagues reported very impressive results for two multiple myeloma patients presenting drug-resistant, disseminated disease [127]. Since MV-NIS is delivered intravenously, the virotherapy is achieved systemically. When treated with MV-NIS, both patients responded to the virotherapy as measured by reduction of M protein levels, and by regression of bone marrow plasmacytosis. Moreover, one of the patients had complete remission. The MV-NIS report serves as a powerful example of how effective virotherapy can be. For the purpose of this review, we will focus on OVs that either represent hallmarks on the history of OV clinical development and/or that more prominently appear to be therapeutically promising.

### 6.1. Adenovirus

ONYX-015 was the first genetically engineered OV to enter clinical trials in 1996. It was designed to selectively replicate in p53-deficient cells through the deletion in the E1B gene [128]. Although a US Phase III randomized trial was planned, clinical development of ONYX-015 was stopped in 2000 after Pfizer acquired Onyx Pharmaceutical’s partner company (San Francisco, CA, USA). Shanghai Sunway Bio Co., Ltd. (Shanghai, China) later purchased the rights to ONYX-015, and the virus was modified with a slightly larger deletion of E1B to create H101. The H101’s Phase III clinical trial randomized 160 Chinese patients with nasopharyngeal cancer to either standard chemotherapy alone (5-FU with cisplatin for treatment naive patients, 5-FU with Adriamycin^®^ (doxorubicin) for previously treated patients), or chemotherapy plus H101. The results showed that the response rate for H101 plus 5-FU and cisplatin was 78.8% versus 39.6% for 5-FU and cisplatin alone, which led to H101 becoming the world’s first OV approved by a regulatory agency [8].

Several modified adenoviruses are currently in clinical trials, including DNX-2401, CG0070, and OBP-301. DNX-2401 is an adenovirus designed to replicate in Rb-deficient cells and contains an RGD (Arg-Gly-Asp) motif to aid in cellular entry via non-CAR receptors [27]. A Phase I trial is currently recruiting participants in order to evaluate efficacy of single-dose DNX-2401 with or without IFN-γ for recurrent glioblastoma and gliosarcoma (NCT02197169). There is a planned Phase II trial to test the efficacy of combination therapy with DNX-2401 and pembrolizumab for recurrent glioblastoma and gliosarcoma (CAPTIVE trial, NCT02798406).

CG0070 is a serotype 5 adenovirus that is also designed to replicate in Rb-deficient cells, but carries the gene for GM-CSF [129]. The CG0070 Phase I clinical trial included 35 bladder cancer patients and demonstrated a complete response after CG0070 treatment in 48.6% of patients with a median duration of complete response of 10.4 months [78]. Currently, both a Phase II (exBOND) and Phase III (BOND2) trial are underway examining CG0070 as a monotherapy in bladder cancer patients who have failed bacillus Calmette-Guerin (BCG) treatment and refuse cystectomy (NCT02143804, NCT02365818 respectively).

OBP-301, a serotype 5 adenovirus designed with a human telomerase reverse transcriptase (hTERT) promoter that drives E1A and E1B expression, is being tested for safety and efficacy as an intratumoral monotherapy for Taiwanese and Korean patients with HCC (NCT02293850) [130].

### 6.2. Herpes Simplex Virus-1

Oncolytic herpes simplex viruses have been engineered to be therapeutically safe, once that HSV is a natural and rather common human pathogen [131]. T-VEC is a modified HSV-1 with deletions in the ICP34.5 and ICP47 genes to decrease neurovirulence and prevent premature viral clearance by abortive apoptosis, respectively. T-VEC also has the therapeutic gene for GM-CSF expression [27]. In a phase III trial compared to GM-CSF alone, T-VEC was shown to significantly improve the durable response rate (16.3% vs. 2.1%, *p* < 0.001), as well as the overall response rate (26.4% vs. 5.7%) in patients with advanced stage melanoma. Overall survival was 23.3 months in the T-VEC arm compared to 18.9 months in the GM-CSF arm (*p* = 0.051) [7]. T-VEC has also been studied in a Phase IB trial in combination with the anti-CTLA4 monoclonal antibody ipilimumab for stage IIIB-IV melanoma. Preliminary results from this study reported an objective response rate of 56%, a durable response rate of 44%, and median progression-free survival of 10.6 months [132]. Given its success and recent FDA approval, several new clinical trials have been initiated using T-VEC. For melanoma, T-VEC is being evaluated as a single-agent treatment in four different trials, including expanded access trials (NCT0214751, NCT02297529) and a Phase II study evaluating the correlation between CD8+ cells and objective response rate after T-VEC treatment (NCT 02366195). There is an ongoing Phase II, multicenter, randomized trial assessing the efficacy and safety of neoadjuvant T-VEC plus surgery compared to surgery alone for resectable stage IIIB-IVM1a melanoma (NCT02211131) [133], as well as a Phase IB/III trial evaluating progression-free survival of T-VEC plus the anti-PD-1 monoclonal antibody pembrolizumab versus pembrolizumab with placebo (NCT02263508). In terms of non-melanoma clinical trials, intratumoral injection of T-VEC is being studied for the treatment of HCC (NCT02509579) and soft tissue sarcoma (NCT02453191).

HF10 is a naturally-occurring, replication-competent HSV-1 with deletions in neurolatency genes UL43, UL49.5, UL55, and UL56. It was initially developed and evaluated for safety and efficacy in Japan [134,135], but has since undergone a Phase I clinical trial in the US. These preliminary results demonstrate HF10 was safe and well-tolerated in both HSV-positive and HSV-negative patients with refractory head and neck tumors or solid cutaneous/superficial malignancies (NCT01017185) [136]. Currently there are two ongoing clinical trials for HF10. The first is a Phase I trial evaluating the safety of repeated dosing of HF10 monotherapy in patients with solid superficial malignant tumors (NCT02428036). The second is a Phase II trial studying the efficacy and safety of HF10 in combination with ipilimumab for Stage IIIB, Stage IIIC, Stage IV unresectable or metastatic melanoma (NCT02272855).

G207, a conditionally-replicative HSV-1 with ICP34.5 deletion and UL39 disruption, was studied in combination with radiotherapy for GBM. Nine patients received a single dose of radiation treatment 24 h after a stereotactically-delivered intralesional dose of G207. Results from this study reported six of nine patients with at least stable disease or partial response with a median survival of 7.5 months after G207 injection (NCT00157703) [119]. Currently, a Phase I trial is recruiting pediatric patients to evaluate safety of G207 as a standalone treatment or with a single dose of radiation therapy in children with progressive or recurrent brain tumors (NCT02457845).

### 6.3. Vaccinia Virus

JX-594 (Pexa-Vec) is a GM-CSF-enhanced vaccinia virus with a TK locus disruption to increase tumor selectivity [18]. Phase I and II trials for JX-594 have been completed in colorectal cancer and HCC, respectively, showing an acceptable safety profile, and demonstrating JX-594 to be overall well tolerated [137,138,139]. As the Phase II randomized study showed improved survival for patients with primary unresectable HCC [138], a Phase III randomized clinical has been initiated to study whether JX-594 followed by sorafenib increases survival compared to sorafenib treatment alone in patients with advanced, chemotherapy-naive HCC (NCT02562755).

GL-ONC1 (GLV-1h68), a luciferase-expressing TK-inactivated vaccinia virus, has been tested in several different tumor models [39,140,141,142]. Preliminary results from two Phase I trials have been reported, one using GL-ONC1 as a standalone treatment for patients with malignant pleural effusions and the other using GL-ONC1 in combination with chemoradiation for head and neck cancer [143,144]. A Phase I trial using this OV for advanced peritoneal carcinomatosis has also been completed (NCT01443260). Currently, there are three ongoing clinical trials using GL-ONC1, including two Phase I trials for patients with refractory ovarian cancer (NCT02759588) and patients with malignant pleural effusions (NCT01766739). The third is a Phase I clinical trial examining the use of GL-ONC1 with or without eculizumab (a monoclonal antibody against complement, which has been suggested to delay OV clearance [145]) prior to surgery in patients with solid tumors who are planned to undergo either curative-intent or palliative-intent surgery (NCT02714374).

### 6.4. Reovirus

Reolysin^®^ is a wild-type reovirus that preferentially infects cells with activated Ras pathways [146]. Phase I data in multiple myeloma patients and advanced solid tumor patients show a favorable safety profile [147,148]. However, Phase II trials have not demonstrated objective responses when Reolysin^®^ was given to patients with metastatic melanoma or in combination with standard chemotherapy for metastatic pancreatic cancer [149,150]. This may be due to the fact that many patients have commonly been exposed to reovirus, and thus have pre-existing reovirus-neutralizing antibodies [27]. Currently there are two active clinical trials for Reolysin^®^: a Phase IB study examining Reolysin^®^ in combination with bortezomib and dexamethasone for relapsed or refractory multiple myeloma (NCT02514382) and a Phase I study for pediatric brain malignancies in combination with GM-CSF (NCT02444546).

### 6.5. Coxsackievirus

Cavatak™ (Coxsackievirus A21) is a wild-type OV that demonstrates natural tropism for malignancies that overexpress ICAM-1 and DAF, the receptors the virus utilizes to infect cells. Results from two recent clinical trials have been reported. Pandha and colleagues reported the results from the Systemic Treatment of Resistant Malignancies (STORM) trial, showing that multiple escalating intravenous doses of Cavatak™ were safe and generally well-tolerated for 30 patients with advanced malignancies [151]. The Phase II trial CAVATAK in Late Stage Melanoma (CALM study) demonstrated an overall response rate of 28.1% and one-year survival of 75.4% for 57 Stage IIIC-IV melanoma patients receiving intralesional injection of Cavatak™ [152]. Like many of the current clinical trials, Cavatak™ trials are exploring the safety and efficacy of combination therapy with other immunotherapies. For advanced melanoma, there two current Phase I trials for intratumoral injection of Cavatak™ in combination with ipilimumab (NCT02307149) and in combination with pembrolizumab (NCT02565992). For bladder cancer, intravesicular Cavatak™ is being tested with or without the addition of low-dose mitomycin C (NCT02316171).

## 7. Future Directions

Since clinicians first noted the oncolytic effect of naturally-acquired viral infection in cancer patients over 100 years ago, significant advances have been made in the understanding of how oncolytic viruses infect and directly kill cells, as well as how they induce a potent antitumor immune response from the host. Given the robust data showing a favorable safety profile in humans and the recent FDA approval of T-VEC for melanoma, OVs are primed to revolutionize the field of cancer treatment. In particular, ongoing clinical trials examining the efficacy of OVs with other immunotherapies should provide valuable insights into ways of further manipulating the body’s immune response to augment OV-directed antitumor responses. At this moment, the lack of side-by-side comparison studies makes it challenging to ascertain which virotherapy, alone or in combination, makes for the best approach in terms of patient outcomes. Studies aimed to determine which cytokines can boost virotherapy are also lacking. Therefore, we can foresee how beneficial such comparison studies will be in guiding not only therapeutic decisions but also the design of OVs. Overall, the future of OVs is highly promising and has offered new hope as a both a curative and adjunctive therapy.

## Figures and Tables

**Table 1:** Features of selected DNA oncolytic viruses.

Virus	Herpes Simplex Virus	Adenovirus	Vaccinia Virus	Parvovirus
Family	Herpesviridae	Adenoviridae	Poxviridae	Parvoviridae
Genome	dsDNA	dsDNA	dsDNA	ssDNA
Method of entry	HVEM, nectin 1 or 2	CAR	Macropinocytosis	Sialic acid residues
Replication site	Nucleus	Nucleus	Cytoplasm	Nucleus
Phase of clinical trials	I–III	I–III	I–III	I–II
Disease types for clinical trials	Melanoma, H and N cancer, pancreatic cancer, GBM, breast cancer, HCC	H and N cancer, pancreatic cancer, GBM, breast cancer, prostate cancer, ovarian cancer, CRC, bladder cancer	H and N cancer, melanoma, lung cancer, breast cancer, HCC, CRC	GBM

Note: dsDNA, double-stranded DNA; ssDNA, single-stranded DNA; HVEM, herpesvirus entry mediator; CAR, coxsackie-adenovirus receptor; H and N, head and neck; GBM, glioblastoma multiforme; HCC, hepatocellular carcinoma; CRC, colorectal cancer.

**Table 2:** Features of selected RNA oncolytic viruses.

Virus	Reovirus	Coxsackievirus	Polio Virus	Seneca Valley Virus	Measles Virus	Newcastle Disease Virus	Vesicular Stomatitis Virus
Family	Reoviridae	Picornaviridae	Picornaviridae	Picornaviridae	Paramyxoviridae	Paramyxoviridae	Paramyxoviridae
Genome	dsRNA	(+)ssRNA	(+)ssRNA	(+)ssRNA	(−)ssRNA	(−)ssRNA	(−)ssRNA
Method of entry	JAM-A	CAR/ICAM1/DAF	CD155	Endocytosis	SLAM, CD46	Endocytosis, direct fusion	LDLR
Replication site	Cytoplasm	Cytoplasm	Cytoplasm	Cytoplasm	Cytoplasm	Cytoplasm	Cytoplasm
Phase of clinical trials	I–II	I–II	I	I–II	I–II	I–II	I
Disease types for clinical trials	H and N cancer, pancreatic cancer, melanoma, ovarian cancer, NSCLC, CRC, glioma, sarcoma	Melanoma, bladder cancer, prostate cancer, breast cancer	GBM	Neuroendocrine tumors, lung cancer, neuroblastoma	Multiple myeloma, ovarian cancer, GBM, oral cancer, peritoneal malignancies	GBM	HCC

Note: dsRNA, double-stranded RNA; ssRNA, single-stranded RNA; JAM-A, junctional adhesion molecule A; CAR, coxsackie-adenovirus receptor; ICAM1, intercellular adhesion molecule 1; DAF, decay-accelerating factor; SLAM, signaling lymphocytic activation molecule; LDLR, low density lipoprotein receptor; H and N, head and neck; NSCLC, non-small cell lung cancer; CRC, colorectal cancer; GBM, glioblastoma multiforme; HCC, hepatocellular carcinoma.
